# Transcriptome Comparison Reveals the Adaptive Evolution of Two Contrasting Ecotypes of Zn/Cd Hyperaccumulator *Sedum alfredii* Hance

**DOI:** 10.3389/fpls.2017.00425

**Published:** 2017-04-07

**Authors:** Qianying Yang, M. J. I. Shohag, Ying Feng, Zhenli He, Xiaoe Yang

**Affiliations:** ^1^Ministry of Education Key Laboratory of Environmental Remediation and Ecosystem Health, College of Environmental and Resources Science, Zhejiang UniversityHangzhou, China; ^2^Department of Agriculture, Bangabandhu Sheikh Mujibur Rahman Science and Technology UniversityGopalganj, Bangladesh; ^3^Institute of Food and Agricultural Sciences, Indian River Research and Education Center, University of FloridaFort Pierce, FL, USA

**Keywords:** comparative transcriptome, SSRs, SNPs, divergent orthologous genes, *Sedum alfredii* Hance, hyperaccumulator, zinc, cadmium

## Abstract

Hyperaccumulating ecotype (HE) and non-hyperaccumulating ecotype (NHE) of *Sedum alfredii* Hance belong to the same species but exhibit contrasting characteristics regarding hyperaccumulation and hypertolerance to cadmium and zinc. The Illumina Hiseq 2500 platform was employed to sequence HE and NHE to study the genetic evolution of this contrasting trait. Greater than 90 million clean reads were obtained and 118,479/228,051 unigenes of HE/NHE were annotated based on seven existing databases. We identified 149,668/319,830 single nucleotide polymorphisms (SNPs) and 12,691/14,428 simple sequence repeats (SSRs) of HE/NHE. We used a branch-site model to identify 18 divergent orthologous genes and 57 conserved orthologous genes of *S*. *alfredii* Hance. The divergent orthologous genes were mainly involved in the transcription and translation processes, protein metabolism process, calcium (Ca^2+^) pathway, stress response process and signal transduction process. To the best of our knowledge, this is the first study to use RNA-seq to compare the genetic evolution of hyperaccumulating and non-hyperaccumulating plants from the same species. In addition, this study made the sole concrete for further studies on molecular markers and divergent orthologous genes to depict the evolution process and formation of the hyperaccumulation and hypertolerance traits in *S*. *alfredii* Hance.

## Introduction

Given long-term evolution and natural selection, a few plants growing in mining soils for a long period of time exhibit strong tolerance and hyperaccumulation of heavy metals within a translocation factor >1. These plants are called hyperaccumulator plants (Baker and Brooks, 1989). Hyperaccumulator plants are ideal candidates for studying heavy metal accumulation mechanisms and remediation of heavy metals/metalloids from contaminated soils (Reeves and Baker, 2000). *Sedum alfredii* Hance is a zinc (Zn)/cadmium (Cd) co-hyperaccumulator. The shoot Zn concentration of *S*. *alfredii* Hance reaches 19,670 mg/kg, and the shoot Cd concentration reaches 9,000 mg/kg (Yang et al., 2002, 2004). However, another ecotype of *S*. *alfredii* is not tolerant to heavy metal, with shoot Zn concentrations of only 490 mg/kg and leaf Cd concentrations of 533 mg/kg (Ni et al., 2004; Xiong et al., 2004).

The difference in heavy metal accumulation ability between the two ecotypes may be attributed to different heavy metal concentrations in the growing environment. Given the strong selective pressure of heavy metal stress and the dominant character of tolerance to heavy metal stress, the selection of tolerance traits between the same species in different growth conditions have become much faster than usual, leading to different tolerance and accumulation of heavy metals (Lefèbvra and Vernet, 1990). The Hyperaccumulating ecotype of *S. alfredii* Hance (HE) was found in an old mining region in Quzhou, Zhejiang Province, China (118°56′ E, 29°17′ N) (Yang et al., 2001, 2002, 2004), which was first mined between the late Ming Dynasty and the early Qing Dynasty (approximately 300 years ago). The identification of plant species was confirmed according to the Arboretum of Zhejiang Province, China (Yang et al., 2002). HE is a new variety of *S. alfredii* Hance given the long-term evolution and natural selection of heavy metals, and it is the nearest relative to the non-hyperaccumulating *S. alfredii* Hance ecotype (NHE) found in the suburb of Hangzhou City (30°06′N, 120°12′E), Zhejiang province. However, genomic variation of the two ecotypes is notable (Chao et al., 2008). Hence, studying the genetic variation between HE and NHE can help understand key processes of heavy metal accumulation and provide a valuable model to investigate the micro-evolution.

To date, only a few researches have been conducted on the genetic variation of hyperaccumulators, especially *S. alfredii* Hance. The Random Amplified Polymorphic DNA (RAPD) method was used to compared the genomic variation of HE, NHE and other four species of *Sedum* and found that HE had ten RAPD bands related to SH containing compounds and resisting osmotic stress (Chao et al., 2008). Up to 2008, eight polymorphic microsatellite markers were developed in HE and NHE, and the average allele number was 5.25 per locus (Huang et al., 2008). However, the information of the evolution of *S. alfredii* Hance is still limited. *Arabidopsis halleri* and *Noccaea caerulescens* are also Zn/Cd co-hyperaccumulator plants. A cross between *A. halleri* and a non-hyperaccumulating, non-tolerant species *A. petraea* was made, and their hyperaccumulation and tolerance characteristics were independent (Macnair et al., 1999). Three quantitative trait loci (QTLs) were identified, representing colocalization with *HMA4* (*Heavy Metal ATPase4*), *MTP1-A* (*Metal Tolerance Protein1-A*), and *MTP1-B* (*Metal Tolerance Protein1-B*) (Willems et al., 2007). The *cis*-regulatory changes and triplication of *AhHMA4* during evolution were the key factors for metal hyperaccumulation of *A. halleri* (Hanikenne et al., 2008). Five *MTP1* paralogues were present in *A. halleri, AhMTP1-A1, -A2, -B, -C*, and *-D* that were the basis of Zn transport and tolerance, but their evolutionary fates were different (Shahzad et al., 2010). The results of the cross between different ecotypes of *N. caerulescens* demonstrated that two QTLs are related to root Zn accumulation (Assuncao et al., 2006), explaining 21.7 and 16.6% of the phenotype variation (Deniau et al., 2006). In addition, three QTLs for Zn and one QTL for Cd accumulation in shoots were also identified. However, in *S. alfredii* Hance, the reproductive organ, stamen and pistil are fused together, making them difficult to emasculate during crossing for QTL analysis. Nevertheless, RNA sequencing is considerably easier and less time consuming; only requiring RNA extraction from tissues.

Comparative transcriptome analysis is a new method to investigate genotypic variation. Using transcript data, we can detect the Simple Sequence Repeat (SSR) molecular marker, which is a convenient tool to study the plant evolution and depict the gene map (Blanca et al., 2011). In addition, single nucleotide polymorphism (SNP) loci and orthologous genes, which can reveal the variance between genotypes, can also be obtained from transcriptome sequences (Novaes et al., 2008; Blanca et al., 2011).

For hyperaccumulator plants, no research is available using a comparative transcriptome to study its genetic evolution. Hence, our research will be the first study to provide ample information of evolution key factors and lay the foundation to study its evolution processes further. In this study, we will employ the comparative transcriptome sequencing method to study the genetic variation of HE and NHE by analysing their SSRs, SNPs and orthologous genes.

## Materials and methods

### Plant growth and treatment

HE was obtained from an old Pb/Zn mining region of Quzhou city in Zhejiang province, and NHE was obtained from a tea plantation of Hangzhou in Zhejiang province. HE plants were grown in non-polluted soils for several generations to reduce the internal metal concentration. We cut the shoots, removed some leaves and cultured them hydroponically. HE and NHE were supplied with basal nutrient solution (Zhang et al., 2016) with or without 100 μM ZnSO_4_, 500 μM ZnSO_4_, or 50 μM CdCl_2_ for 7 days. The pH of the nutrient solution was adjusted to 5.8 every other day. The nutrient solution was continuously aerated and renewed every 3 days. The plants were grown in a growth chamber with a 16/8-h photoperiod at 400 μM m^−2^ s^−1^, day/night temperatures of 26°/20°C, and humidity of 70/85%. Three biological replicates were performed for each treatment. The upper shoots and new roots were harvested separately for transcriptome analysis and placed in liquid nitrogen immediately.

### Libraries establishment, illumina sequencing, *de novo* assembly and annotation

Total RNA was extracted from HE and NHE shoots and roots with RNAout kit (Tiangen, China). First, we assessed the degradation and contamination of RNA on 1% agarose gels. The purity of RNA was assessed using the NanoPhotometer® (IMPLEN, CA, USA), and the concentration of RNA was measured using Qubit® RNA Assay Kit in Qubit® 2.0 Flurometer (Life Technologies, CA, USA). RNA integrity was assessed using the RNA Nano 6000 Assay Kit of the Agilent Bio-analyzer 2100 system (Agilent Technologies, CA, USA). A total amount of 1.5 μg of qualified RNA per sample was used to establish the HE and NHE libraries, separately. Sequencing libraries were generated using the NEBNext® Ultra™ RNA Library Prep Kit for Illumina® (NEB, USA). Briefly, mRNA was purified using magnetic beads and divalent cations under elevated temperature. First strand cDNA was synthesized using random hexamer primers and RNase H. Second strand cDNA synthesis was performed using DNA polymerase I and RNase H. The library fragments were purified with AMPure XP system (Beckman Coulter, Beverly, USA) to select cDNA 150–200 bp in length. Library quality was assessed on the Agilent Bioanalyzer 2100 system (Agilent Technologies, CA, USA).

HE and NHE library preparations were sequenced on an Illumina Hiseq 2500 platform and 125-bp paired-end reads were generated. First, raw data in the fastq format were processed, and clean data were obtained using Trimmomatic (Lohse et al., 2012) by removing reads containing adapter, reads containing ploy-N and low quality reads from raw data. All the downstream analyses were based on clean data with high quality. HE and NHE sequences were assembled separately. Transcriptome assembly was accomplished based on the left files and the right files using Trinity (Grabherr et al., 2011) with min_kmer_cov set to 2 by default, and all other parameters were set to default. The longest transcripts of each gene were chosen as unigenes. The length of transcripts and unigenes were calculated.

The unigenes were annotated based on the following seven databases: Nr (NCBI non-redundant protein sequences), Nt (NCBI non-redundant nucleotide sequences), Pfam (Protein family), KOG (eukaryotic Ortholog Groups), Swiss-Prot (A manually annotated and reviewed protein sequence database), KO (KEGG Ortholog database) and GO (Gene Ontology). The e-values cut-off of NR, NT and Swiss-Prot were 1e-5, and the e-value cut-off of KOG was 1e-3 using NCBI blast 2.2.28+. The e-values cut-off of KEGG, Pfam and GO were 1e-10, 0.01 and 1e-6, using KAAS, hmmscan and blast2go, respectively. According to the GO annotation, the unigenes of each ecotype were classified into biological process, cell component and molecular function. Based on the KOG annotation, the unigenes of HE and NHE were classified into 26 groups, respectively. The number of unigenes of HE and NHE under the second level of KEGG ortholog were calculated. The CDS detection followed the following two steps: (i) The unigenes were blast against the Nr and Swiss-port databases, and an open-reading frame (ORF) was extracted and translated into a peptide sequence; (ii) The unigenes without align results or hits against the not-predicted sequences would be predicted the ORF and translated into peptide sequence with the standard codon table using ESTSCAN software (3.0.3).

### SNPs calling and SSRs detection

Picard - tools v1.41 and samtools v0.1.18 were used to establish the index of referenced transcriptome assembly sequence. The clean reads of each sample obtained from HE or NHE were mapped to transcriptome assembly sequence of HE or NHE to generate the bam files. The ReorderSam.Jar tool implemented in Picard—tools was used for hierarchical ranking for bam files. SortSam.Jar implemented in Picar—tool was used to sort and AddOrReplaceReadGroups. Jar was used to add identified information to bam file to fulfil1 the file format for GATK variation detection. SNP calling was performed by GATK2 (McKenna et al., 2010) software. Raw vcf files were filtered with the GATK standard filter method and other parameters (clusterWindowSize: 10; MQ0 ≥ 4 and (MQ0/ (1.0*DP)) > 0.1; QUAL < 10; QUAL < 30.0 or QD < 5.0 or HRun > 5), and only SNPs with quality > 30 and distance > 5 were retained.

SSRs of the transcriptome were identified using MISA (http://pgrc.ipk-gatersleben.de/misa/misa.html) with the default parameters, and the minimum repeats of mono-, di-, tri-, tetra-, penta-, and hexanucleotide motifs were 10, 6, 5, 5, 5, and 5, respectively. The distribution densities of different SSRs types in transcriptome were calculated.

### Identification of orthologous genes

The CDSs of each unigene were extracted and translated into amino acid sequences. Blast-based approach performs all-to-all BLAST before ortholog classification. All-to-all BLASTP was conducted for all amino acid sequences with a cut-off e-value of 1e−5. Orthologous groups were constructed from the BLASTP results using OrthoMCL v2.0.3 (Li et al., 2003) with default settings. The CDS of *Vitis vinifera* was used as an internal reference, and the CDS of *A. thaliana* was used as an external reference. The orthologous groups sequences were one-to-one blasted against the CDS of *V. vinifera* and *A. thaliana* using Muscle 3.8.31. The Ka/Ks ratios, which indicate the ratios of non-synonymous rate (Ka) to synonymous rate (Ks), were calculated by PAML (Yang, 2007). The non-synonymous rate indicates the number of non-synonymous substitutions per non-synonymous site. The synonymous rate indicates the number of synonymous substitutions per synonymous sites. Synonymous refers to the nucleotide substitutions that do not change the encoded amino acid, and non-synonymous refers to the nucleotide substitutions that change the amino acid. We used a branch-site model (model = 2, Nsites = 2) in the codeml program in the PAML package to detect the signatures of selection on individual codons in a specific branch (Zhang et al., 2005). The HE branches were set as the foreground branch, and the optimized branch-site model was used. A likelihood ratio test (LRT) compared a model with positive or purified selection on the foreground branch with a null model in which no significant selection occurred on the foreground branch and calculated the statistic to obtain a *P*-value. With *P*-values less than 0.05, the genes with Ka/Ks > 1 were identified as divergent orthologous genes, which evolve under positive selection. Genes with Ka/Ks < 0.1 were identified as conserved orthologous genes that were purified under heavy selection constraint likely due to a conserved function. The orthologous genes with 0.1 < Ka/Ks < 1 were identified as neutral orthologous genes. The analysis logic flow is presented in Figure S1 online.

### KEGG pathway enrichment of orthologous genes

The divergent and conserved orthologous genes were annotated based on seven databases. KOBAS (Mao et al., 2005) software was used to test the statistical enrichment of divergent and conserved genes ortholog groups in KEGG pathways. The *P*-values in the significance tests (corrected *P*-value < 0.05) were corrected via false discovery rate (FDR).

### Sequencing confirmation

To confirm the accuracy of the Illumina Hiseq 2500 platform sequence and Trinity assembly, we randomly chose 15 divergent orthologous genes from both HE and NHE, and resequenced these genes using Sanger sequencing. The primers were designed using Primer 3 (Koressaar and Remm, 2007). We used 55°C as the annealing temperature, and sequencing was conducted with an ABI BigDye® Terminator kit on an ABI 3730 DNA Analyzer. Primer information is presented in Table S1.

### Heavy metal analysis

The remaining shoots and roots were used to determine Zn and Cd concentration by Inductively Coupled Plasma Mass Spectroscopy (ICP-MS) (Agilent, USA). We used GBW10014 (GSB-5) CRM Cabbage as an internal reference. The Zn concentration detected in CRM Cabbage was 27.23 mg/kg, which was in the range of the standard value of 26 ± 2 mg/kg, and the Cd concentration detected in CRM Cabbage was 31 μg/kg, which was in the range of the standard value of 35 ± 6 μg/kg. The heavy metal concentration was statistically analyzed using student's *t*-test and ANOVA.

## Results

### Transcriptome sequence and assembly

**Figure 1:**
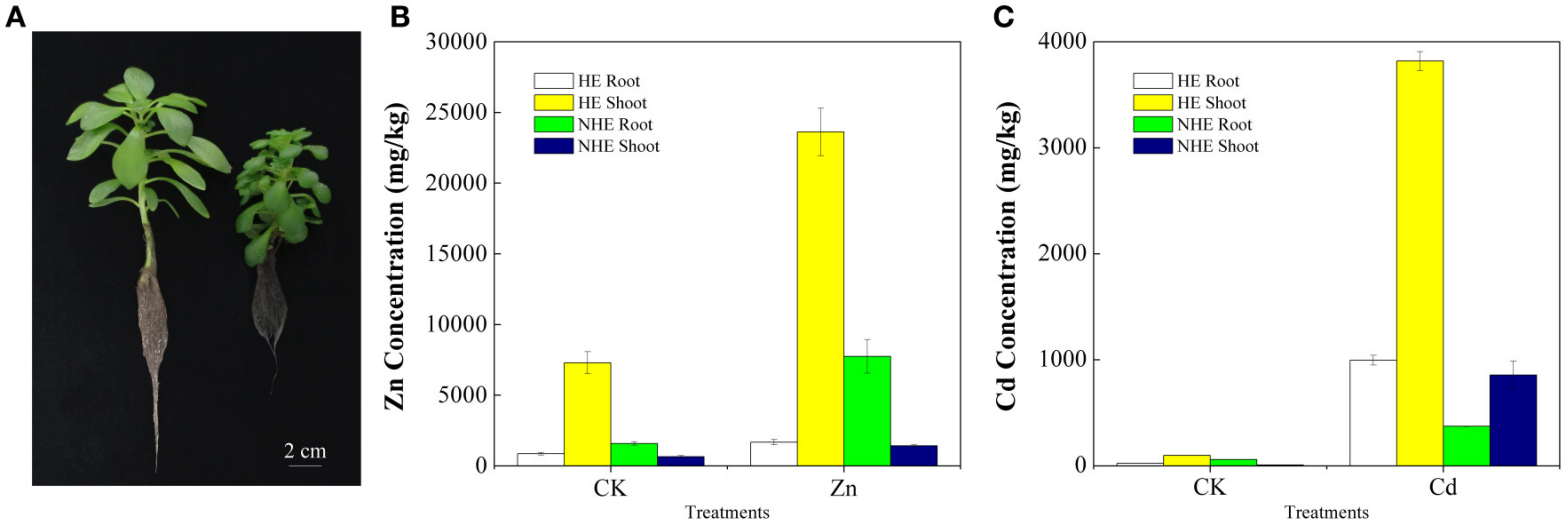
**The phenotypes of hyperaccumulating ecotype (HE) (left) and non-hyperaccumulating ecotype (NHE) (right) of ***Sedum alfredii*** Hance (A)** and Zn concentration **(B)** and Cd concentration **(C)** of *Sedum alfredii* Hance. Control treatment was only nutrient solution. Zn treatment was 100 μM ZnSO_4_ and Cd treatment was 50 μM CdCl_2_.

The morphological phenotypes of HE and NHE are comparable on normal growth conditions (Figure 1A). When supplied with 100 μM Zn or 50 μM Cd, the HE plants did not exhibit any toxicity symptoms with green leaves and white roots. However, the NHE plants exhibited severely toxic symptoms with stale leaves and brown roots. HE accumulated greater than 20,000 mg/kg Zn in shoots when supplied with 100 μM Zn for 7 days, and its shoots also accumulated more than 3,500 mg/kg Cd when supplied with 50 μM Cd. However, NHE accumulated only 1,422 mg/kg Zn or 857 mg/kg Cd in shoots when supplied with 100 μM Zn or 50 μM Cd. In addition, Zn was mainly stored in roots of NHE, whereas HE, Zn and Cd are mainly transported to shoots (Figures 1B,C).

To obtain sequences of a large number of expressed genes, we used the RNA of shoots and roots of HE and NHE plants supplied with 0 μM, 100 μM Zn, 500 μM Zn, and 50 μM Cd for sequencing on the Illumina Hiseq 2500 platform. We obtained 94,134,864 and 103,397,468 raw reads of HE and NHE, respectively (Table 1). After cleaning the raw reads with adaptor related, with containing poly-N and low quality, we obtained 92,371,082 clean reads in total for both ends with 125 bp of HE, which was equal to 11.54 G. We obtained 101,217,674 clean reads for each end with 125 bp of NHE, which was equal to 12.66 G (Table 1). After assembling the clean reads of HE and NHE separately, 157,226 transcripts with a mean length of 846 bp of HE and 269,592 transcripts with a mean length of 658 bp of NHE were obtained (see Table 1 and Figure S2 online). We chose the longest transcript of each gene as the unigene. In total, we acquired 118,479 unigenes with a mean length of 609 bp for HE and 228,051 unigenes with a mean length of 518 bp for NHE (Table 1). Ranking the transcripts from long to short and summing the length of the transcripts, the contig N50 is the length of the smallest contig in the set that contains the fewest (largest) contigs whose combined length represents at least 50% of the assembly, while the N90 is the length of the smallest contig in the set that contains the fewest (largest) contigs whose combined length represents at least 90% of the assembly (Miller et al., 2010). The N50 and N90 values of unigenes of HE were 1,009 and 244, respectively, whereas the N50 and N90 values of unigenes of NHE were 656 and 235, respectively (Table 1). The length distribution of transcripts and unigenes indicated that the <301 size class constituted most transcripts and unigenes, and the number of transcripts and unigenes decreased as length size increased (see Figure S2 online). All raw data and clean data were deposited in the NCBI Sequence Read Archive repository (Accession Number: SRR5061928 for HE *S. alfredii* Hance and SRR5082565 for NHE *S. alfredii* Hance).

**Table 1:** **Basic information of raw reads, clean reads, assembled unigenes, annotation, CDS, SNP, and SSR loci**.

**Species**	**No. of raw reads**	**No. of clean reads**	**No. of unigenes**	**N50/N90 value of unigenes (mean length)**	**Annotation (percentage)**	**CDS**	**SNP**	**SSR**
HE	94,134,864	92,371,082	118,479	1,009/244 (609)	73,617 (62.13%)	109,685	149,668	12,691
NHE	103,397,468	101,217,674	228,051	656/235 (518)	149,378 (65.5%)	214,358	319,830	14,428

*HE, Hyperaccumulating ecotype; and NHE, non-hyperaccumulating ecotype of Sedum alfredii Hance*.

To confirm the accuracy of the Illumina sequences and Trinity assembly, we used Sanger sequencing to re-sequence divergent orthologous genes, including six HE fragments and nine NHE fragments. For HE, the total length was 2,409 bp, and only three sites were different, indicating that the consistency rate was 99.9%. For NHE, the total length was 3,928 bp, and the consistency rate was 99.7% within 12 different sites (see Table S1 online). The high consistency rates of HE and NHE confirmed the accuracy of Illumina sequence and Trinity assembly.

### Unigenes of HE and NHE annotation

A total of 73,617 (62.13%) unigenes of HE and 149,378 (65.5%) unigenes of NHE were successfully annotated in at least one of the seven databases: Nr, Nt, Pfam, KOG, Swiss-Prot, KO and GO (Table 1, Table S2). The highest annotation rate of the database was Nr, in which 52.61% of unigenes of HE and 55.66% of unigenes of NHE were annotated. We extracted 109,685 coding region sequences (CDS) of HE and 214,358 CDS of NHE from blast or ESTSCAN results (Table 1).

**Figure 2:**
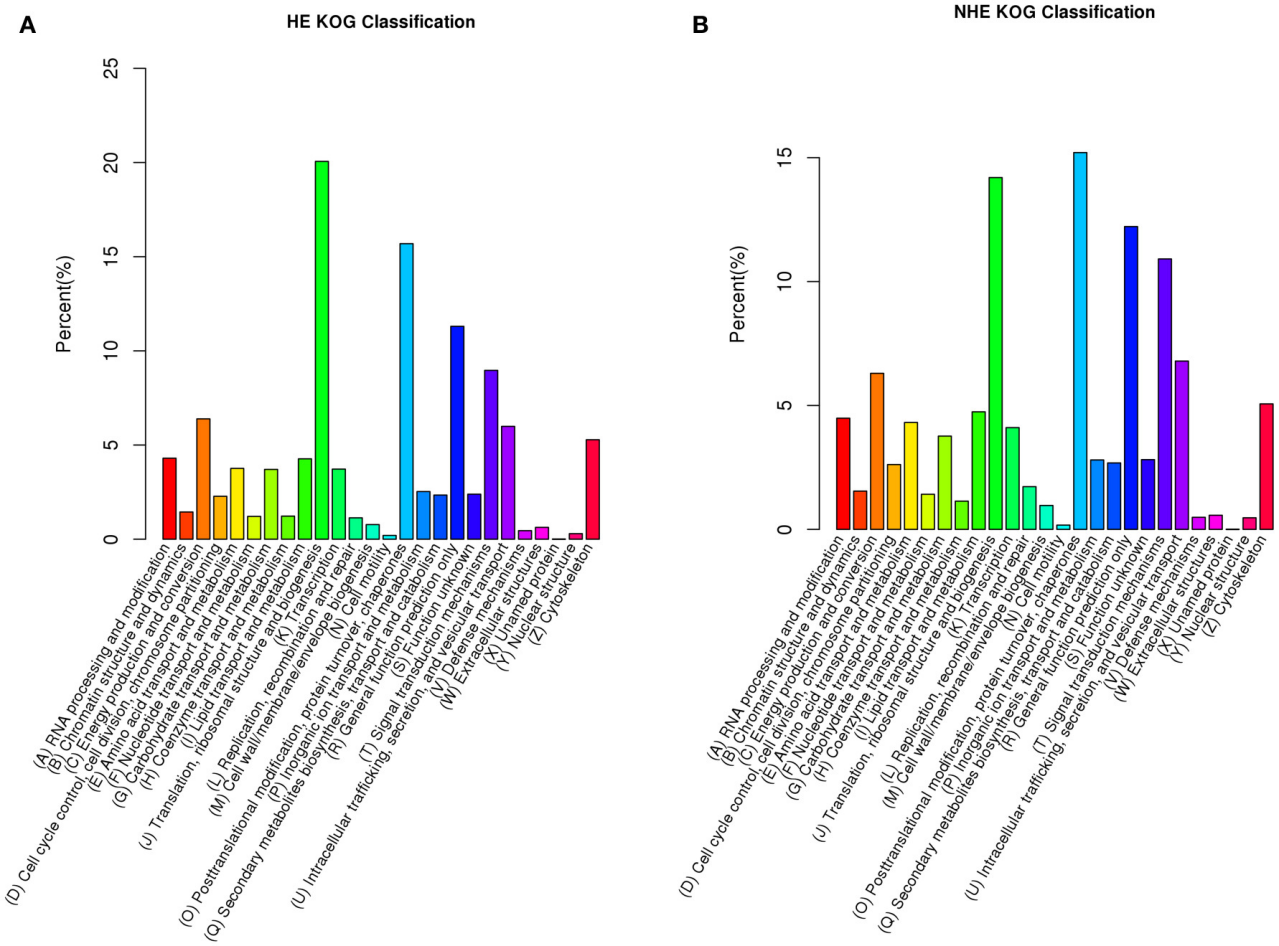
**Unigenes annotation based on eukaryotic Ortholog Groups (KOG) of hyperaccumulating ecotype (HE) (A)** and non-hyperaccumulating ecotype (NHE) **(B)** of *Sedum alfredii* Hance.

The major KOG group of HE was group (J): translation, ribosomal structure and biogenesis (20.1%) (Figure 2A); the major KOG group for NHE was group (O): posttranslational modification, protein turnover and chaperones (15.2%) (Figure 2B). The percentages of other groups for HE and NHE were similar. A total of 54,994 unigenes of HE and 104,365 unigenes of NHE were annotated in GO (see Table S2 online). GO is classified into three ontologies, including biological process, cellular component, and molecular function. HE and NHE represented the same patterns of GO annotation. The major biological process terms were the cellular process, metabolic process and single-organism process. The major cellular component terms included the cell, cell part, macromolecular complex and organelle. The major molecular function terms were binding and catalytic activity (see Figure S3 online). There were 28,150 unigenes of HE and 48,697 unigenes of NHE annotated in KEGG ortholog (see Table S2 online). The major KO group for both HE and NHE was translation, and the second major KO group was signal transduction (see Figure S4 online). The results of KOG annotation and KEGG ortholog annotation suggested that expression regulation plays a significant role in hyperaccumulation traits of *S. alfredii*.

### SNPs and SSRs analysis

We identified 149,668 SNPs loci of HE and 319,830 SNPs loci of NHE (Table 1). Among them, A/G was the most frequent SNP type, occupying 30.2 and 30.9% of HE and NHE, respectively, whereas C/T was the second most frequent SNP type, occupying 29.3 and 30.4% of SNP types of HE and NHE, respectively (see Table S3 online). The percentages of non-coding regions SNPs sites of HE and NHE were 63.17 and 53.36%, respectively (see Table S4 online). The percentages of non-synonymous SNPs were 0.11 and 0.09% for HE and NHE, respectively (see Table S4 online), indicating that the functional mutation rate was very low. The detailed SNPs information for HE and NHE were available from Datasheets S1, S2.

We identified 12,691 SSRs of HE and 14,428 SSRs of NHE (Table 1). For HE, 10,746 unigenes contained SSRs, and 1,579 unigenes contained more than one SSR. A total of 5.0% SSRs were characterized as compound forms. Among the SSRs, the most abundant repeat unit type was monomers (39.7%), followed by trimers (38.4%), dimers (20.0%), tetramers, hexamers and pentamers (see Table S5 online). For NHE, the assay revealed that 12,393 unigenes contained SSRs, and 1,653 unigenes contained more than one SSRs. In total, 5.3% SSRs were characterized as compound forms. Among the SSRs, the most abundant repeat unit type was trimers (43.4%), followed by monomers (34.0%), dimers (19.6%), tetramers, hexamers and pentamers (see Table S4 online). Both for HE and NHE, the most abundant repeat type of monomers were 9–12 repeats, and the other most abundant repeat type of other motifs were 5–8 repeats (see Figure S5 online). The detailed repeat type frequency for HE and NHE were available from Datasheet S3.

### Orthologous genes analysis

**Figure 3:**
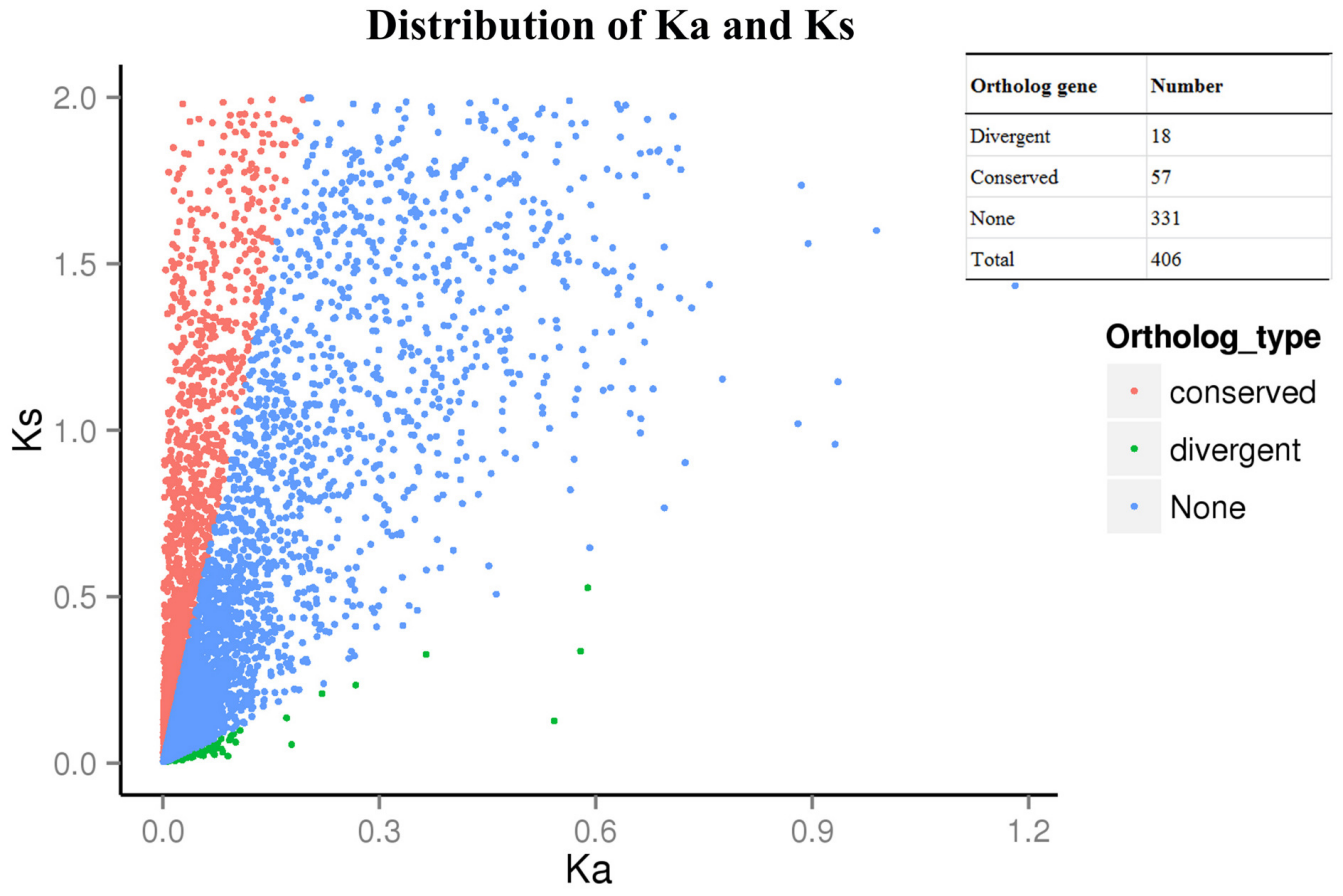
**Distribution of Ka and Ks. Ka/Ks>1 as divergent ortholog gene, Ka/Ks<1 as conserved ortholog gene and 0.1<Ka/Ks<1 as noneortholog gene**.

**Figure 4:**
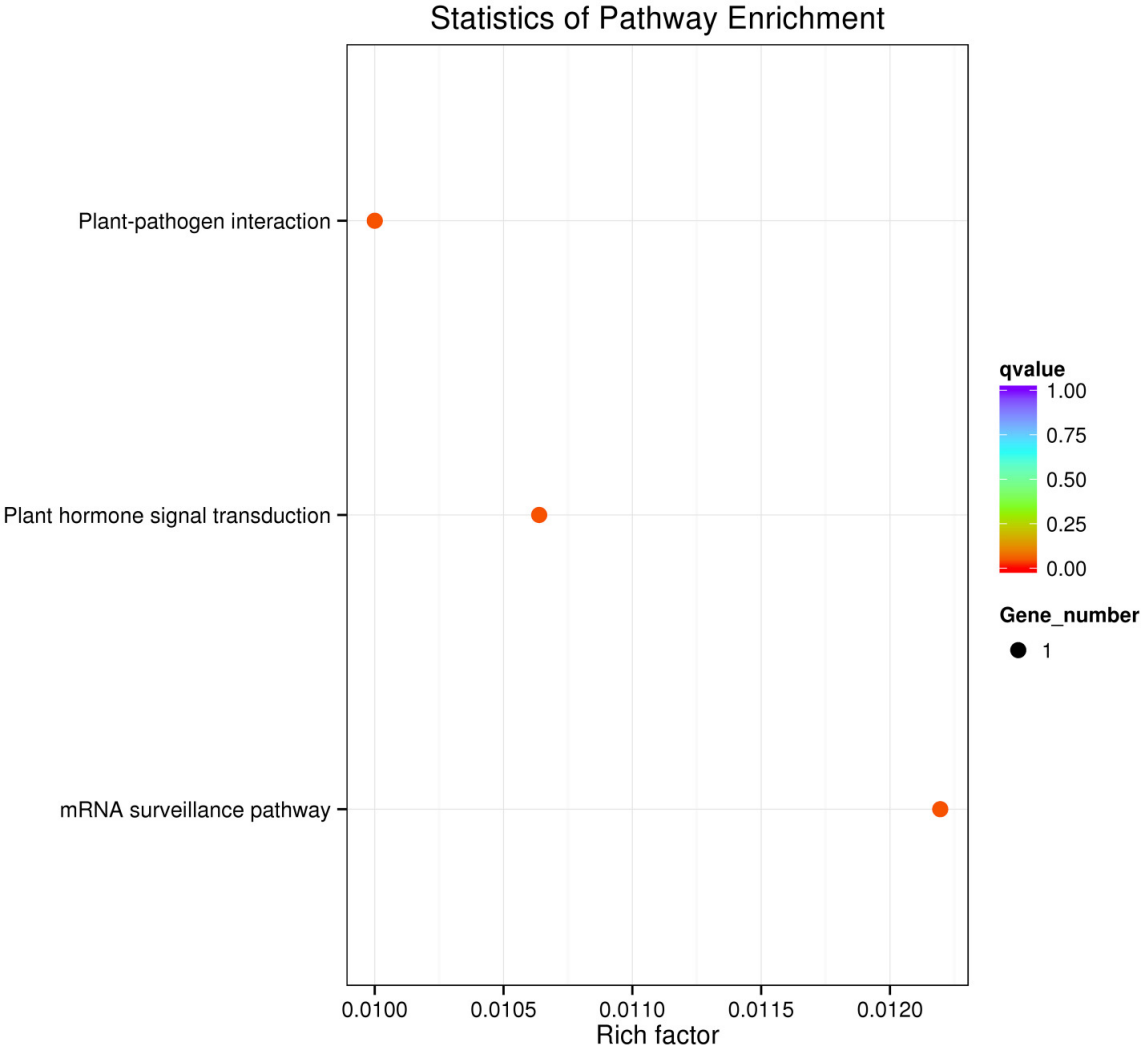
**Unigenes Kyoto Encyclopedia of Genes and Genomes (KEGG) of divergent orthologous genes**.

After using the branch-site model and likelihood ratio test, 18 divergent orthologous genes, 57 conserved orthologous genes and 331 none (neutral) orthologous genes were identified (Figure 3). The annotation results of divergent orthologous genes indicated that there were two 28S ribosomal RNA genes, two calmodulins, two histidine kinases, two nuclear transcription factor Y genes, and two WRKY transcription factors, and the other orthologous genes were apolipoprotein, calcipressin, cysteine proteinase inhibitor, F-actin capping protein, proteasome, receptor-like protein kinase, RNA polymerase C-terminal repeat and vacuolar-sorting receptor (Table 2). KEGG pathway enrichment indicated that only three genes were significantly enriched in the KEGG pathway, including plant-pathogen interaction, plant hormone signal transduction and mRNA surveillance pathway (Figure 4), implying that these pathways may evolve under heavy metal stress.

**Table 2:** **Information and annotation of divergent orthologous genes**.

**OG ID**	**Contig ID**	**Contig ID**	**Ka**	**Ks**	**Ka/Ks**	***p*-value**	**Functional annotation**
OG30580	HE|c48427_g1	NHE|c76500_g6	0.1715	0.1358	1.2634	0.010034	RNA polymeraseII Rpb1 C-terminal repeat
OG30137	HE|c44762_g1	NHE|c66407_g1	0.0485	0.0285	1.7055	0.002957	28S ribosomal RNA gene
OG11524	HE|c41849_g1	NHE|c75692_g1	0.026	0.0201	1.2922	0.023937	28S ribosomal RNA gene
OG30653	HE|c4929_g1	NHE|c62840_g1	0.0901	0.0213	4.2322	0.007088	Proteasome
OG29785	HE|c42238_g2	NHE|c75239_g2	0.0704	0.031	2.2756	0.006675	Cysteine proteinase inhibitor
OG34157	HE|c87800_g1	NHE|c56126_g1	0.0378	0.0329	1.1484	5.73E-05	Calmodulin
OG27602	HE|c24377_g1	NHE|c218476_g1	0.3649	0.3268	1.1166	0.005169	Calmodulin
OG26704	HE|c12628_g1	NHE|c149750_g1	0.1785	0.0557	3.2028	0.003417	Calcipressin
OG27764	HE|c26038_g1	NHE|c62425_g1	0.5789	0.3365	1.7202	0.029386	Nuclear transcription factor Y
OG30430	HE|c47086_g2	NHE|c56724_g1	0.0536	0.0476	1.1268	0.024629	Nuclear transcription factor Y
OG31192	HE|c54459_g1	NHE|c6500_g1	0.5425	0.127	4.2702	8.39E-07	WRKY transcription factor
OG04684	HE|c107180_g1	NHE|c62757_g1	0.0223	0.0159	1.4035	0.010898	WRKY transcription factor
OG30906	HE|c51477_g2	NHE|c74553_g1	0.083	0.0328	2.5294	0.003113	Apolipoprotein
OG31045	HE|c52729_g3	NHE|c53528_g1	0.0563	0.0226	2.4965	0.000715	Histidine kinase
OG27948	HE|c28007_g1	NHE|c69277_g1	0.042	0.0192	2.1828	0.003796	Histidine kinase
OG33400	HE|c78721_g1	NHE|c69638_g1	0.0629	0.04	1.571	0.001293	Receptor-like protein kinase
OG23769	HE|c40589_g1	NHE|c65962_g1	0.0714	0.0254	2.8066	0.016308	F-actin capping protein
OG31469	HE|c56909_g1	NHE|c61038_g1	0.0271	0.0091	2.9802	0.005782	Vacuolar-sorting receptor

*HE, Hyperaccumulating ecotype; and NHE, non-hyperaccumulating ecotype of Sedum alfredii Hance*.

In total, 13 (22.8% of all conserved orthologous genes) conserved orthologous genes were annotated as ribosomal proteins, including four 40S ribosomal proteins, one 50S ribosomal protein and eight 60S ribosomal proteins (see Table S6 online). There were no conserved orthologous genes statistically significantly enriched in KEGG pathway, but relative enrichment terms were ribosome, cell processes including protein processing in the endoplasmic reticulum, phagosome, and endocytosis. The conserved orthologous genes were also relatively enriched in some primary metabolic pathway, such as glutamate metabolism, glyoxylate and dicarboxylate metabolism, nitrogen metabolism fructose and mannose metabolism and citrate cycle (TCA cycle) (see Figure S6 online), indicating that ribosomes and primary metabolic pathways were conserved through genetic evolution.

## Discussion

HE plants grow in an old Zn/Pb mining region, whereas NHE plants grow in a tea plantation in Hangzhou. Both HE and NHE belong to the same species but have contrasting abilities to accumulate and tolerate Cd, Zn and Pb based on their different growing environments. The occurrence of different adaptation abilities to heavy metals in 65 populations of *A. halleri* in Europe was also attributed to geographical isolation (Pauwels et al., 2012). Hence, the environmental pressures of the Zn/Pb mining region forced the HE ecotype to evolve characteristics from NHE to adapt to heavy metal stress. In this study, we provided genetic information for both HE and NHE and revealed the variation between them, which can compensate for the gap of knowledge in molecular markers (SSRs and SNPs) and orthologous genes of *S. alfredii* Hance.

For GO annotation, the metabolic process, regulation of biological process and response to stimulus of biological process were enriched, which was consistent with the previous results of *S. alfredii* shoot Cd transcriptome analysis (Gao et al., 2013). In addition, the extracellular region and membrane of cellular component items, catalytic activity, structural molecular activity and transporter activity of molecular function were also enriched, which was similar with the GO enrichment results of a previous study (Gao et al., 2013), suggesting that the Cd accumulation ability of HE may be related to the genetic evolution of HE. The zinc transcriptome of hyperaccumulator *Populus* × *euramericana* leaves also revealed that the structural molecule activity, receptor activity, transporter activity and enzyme regulator activity were enriched in GO (Di Baccio et al., 2011), which was similar to our results. In addition, antioxidant activity was enriched for *S. alfredii* and *N. caerulescens* (Halimaa et al., 2014), suggesting that the genes in these GO categories may play roles in hyperaccumulation or hypertolerance to heavy metals.

A large amount of SSR and SNP markers boosted population transcriptome approaches to characterize the evolution process and provide signatures of selection (Stinchcombe and Hoekstra, 2008; Siol et al., 2010). Nevertheless, no SNP resources have been previously reported for *S. alfredii*. Here, we reported 149,668 SNPs for HE and 319,830 SNPs for NHE (Table 1), providing novel genomic markers to characterize the polymorphism diversity of *S. alfredii*. In a previous study, eight genomic SSRs were identified as polymorphic between HE and NHE leaves (Huang et al., 2008), and 6,176 perfect SSRs and 3,019 imperfect SSRs were also found in HE shoots (Gao et al., 2013). In this study, we identified 12,691 SSRs for HE and 14,428 SSRs for NHE from both shoots and roots (Table 1), providing more complete microsatellite information for further molecular population genetic studies.

To determine how HE *S. alfredii* Hance adapt to environments with heavy metal stress is important for research in the evolutionary biology of hyperaccumulators. The ratio of Ka and Ks has been widely used for the deduction of evolutionary dynamics and indication of adaptive symbols of protein-coding sequences (Yang and Bielawski, 2000). The orthologous genes with a Ka/Ks ratio greater than 1 were highly diverged under the natural selection pressure. Given that we combined all the RNA samples together to establish libraries and did the analysis, we did not examine the orthologous gene expression level. In the present study, we identified 18 divergent orthologous genes between HE and NHE. We classified them into five groups: transcription and translation processes, protein metabolism process, calcium (Ca^2+^) pathway, stress response process and signal transduction process (Table 2).

The RNA polymerase II Rbpl C-terminal repeat (OG30580) and 28S ribosomal RNA gene (OG30137 and OG11524) were related to transcription and translation processes. RNA polymerase II transcribes all protein-encoding genes into mRNA, non-coding RNAs, small nucleolar RNAs and microRNAs (Cramer et al., 2008). Its largest subunit Rpb1 evolved a repetitive carboxy-terminal domain (CTD) (Egloff and Murphy, 2008), of which phosphorylation status determine the activities of RNA polymerase II complex and associated proteins (Hajheidari et al., 2013). The CTD phosphatases are involved in ABA signaling, normal growth and development (Bang et al., 2006) and xenobiotic detoxification pathways in plants (Fukudome et al., 2014). The 28S ribosomal RNA gene is part of the rRNA transcriptional unit (Long and Dawid, 1980), and its divergent regions known as “D”-regions are typically used as a phylogenetic marker for analysing evolution (Gou et al., 2013). The genes involved in transcription and translation processes diverging under evolution suggested that *S. alfredii* evolved variations in fundamental biological processes under heavy metal stress.

Proteasome (OG30653) and cysteine proteinase inhibitor (OG29785) belonged to protein metabolism process. When plants suffer from heavy metal stress, such as from Cd, arsenic (As) and chromium (Cr), damaged proteins resulting from oxidative stress are produced. The ubiquitin/proteasome 26S system is subsequently activated to degrade and remove the damaged proteins (Dametto et al., 2015). Cysteine proteases will lead to inappropriate proteolysis in high concentrations, so cysteine protease inhibitors are needed to correctly and appropriately regulate enzymatic activity (Bobek and Levine, 1992). Previous studies demonstrated that cysteine proteinase inhibitors increase tolerance to salt, drought, oxidation, and cold in plants (Zhang et al., 2008; Li et al., 2015). Cysteine proteinase inhibitors also interact with a calcium/calmodulin-binding receptor-like kinase or a Ca^2+^-dependent nuclease to regulate plant alkaline stress tolerance (Sun et al., 2014).

In our study, calmodulin (OG34157 and OG27602) and calcipressin (OG26704) were divergent orthologous genes. In addition, whether cysteine proteinase inhibitors also interact with proteins regulated by calcipressin or calmodulin to form hypertolerance mechanisms responding to heavy metal stress should also be assessed. Calmodulin senses the intracellular Ca^2+^ concentration and regulates a highly conserved type 2B protein phosphatase, calcineurin, which is vital for mediating cellular stress responses (Aramburu et al., 2004). Calcineurin activity is also regulated by calcipressins positively or negatively (Davies et al., 2007). In our previous study, we demonstrated that exogenous Ca^2+^ inhibited Cd influx into roots (Lu et al., 2010) and significantly increased glutathione biosynthesis to alleviate growth inhibition from Cd stress (Tian et al., 2011b) in *S. alfredii* Hance. The distribution pattern of Cd is similar to Ca in the leaves of HE but not in NHE, suggesting that the Ca pathway may be associated with Cd detoxification (Tian et al., 2011a) and Cd^2+^ may compete with Ca^2+^ for calmodulin binding (Rivetta et al., 1997).

Nuclear transcription factor Y (NF-Y) (OG27764 and OG30430), WRKY transcription factor (OG31192 and OG04684) and apolipoprotein (OG30906) belong to the stress response process. NF-Y binding to the CCAAT box is ubiquitous in plants and is emerging as a significant regulator of the stress-induce response (Leyva-Gonzalez et al., 2012). NF-Ys are highly induced in response to low phosphorus, low nitrogen, high salinity, oxidative, heat and drought stress (Li et al., 2008; Hackenberg et al., 2012; Leyva-Gonzalez et al., 2012; Xu et al., 2014). WRKY transcription factors recognize the W-box of target genes and induced their expression to mediate abiotic stress (Phukan et al., 2016). Previous studies demonstrated that WRKYs were up-regulated under Cd stress in *A. thaliana, N. caerulescens, Tamarixhispida*, and *Populussimonii* × *Populusnigra* (Wei et al., 2008; Opdenakker et al., 2012; Zhao et al., 2015; Yang et al., 2016). It was found that WRKY transcription factors are also modulated in response to Pi homeostasis, iron starvation, and cold stress in rice (Dai et al., 2016). Apolipoprotein is involved in modulating tolerance to oxidative stress, freezing, heat shock, and paraquat treatment (Charron et al., 2002, 2008). The significant divergence of these transcription factors may be involved in the heavy metal stress response in *S. alfredii* Hance.

We classified histidine kinase (OG31045 and OG27048) and receptor-like protein kinase (OG33400) into the signal transduction process group. Histidine kinase (HK) operates through the cytokinin signal transduction pathway and controls numerous physiological processes in plants (Narusaka et al., 2004). Recently, AHK5 was reported to play roles in maintaining the H_2_O_2_ homeostasis, perceiving nitric oxide and ethylene signals (Desikan et al., 2008). In addition, numerous histidine kinases are involved in drought, salt, cold, and osmotic stress responses (Urao et al., 1999; Tran et al., 2007; Jeon et al., 2010; Pham et al., 2012). Receptor-like protein kinase forms the largest group of eLRR-containing cell surface receptors (Shiu and Bleecker, 2003), which perceive extracellular signals at the plasma membrane (Walker and Zhang, 1990) and play roles in abscisic acid signaling, disease resistance and fungal pathogens resistance (Komjanc et al., 1999; Fritz-Laylin et al., 2005; Wang et al., 2008; Cova et al., 2010). The divergence of signal perception genes suggested that HE may evolve a more sensible signal perception system in response to heavy metal influx into roots. Further studies on relative expression levels of signal transduction genes and protein-protein interaction assay by yeast two-hybrid analysis and bimolecular fluorescence complementation assay will verify this hypothesis.

## Conclusion

Altogether, this study was the first to use comparative RNA-sequencing technology to depict the evolution variation of hyperaccumulator plants. We obtained large amounts of sequence information from shoots and roots of HE and NHE. We identified molecular markers, such as SNPs and SSRs of *S. alfredii* Hance, which are an important resource for research in hyperaccumulation and hypertolerance traits in genome mapping. The 18 divergent orthologous genes were mainly involved in transcription and translation processes, protein metabolism process, Ca^2+^ signaling pathway, stress response process and signal transduction process, which are key factors for HE in adaptive evolution to heavy metal-contaminated environments. In general, our results may lay a solid foundation for research into molecular systematic, population genetic and evolution processes of *S. alfredii* Hance.

## Author contributions

XY, QY, and MS designed the experiment. QY and MS performed the experiment. QY analyzed the data of the work. QY wrote the manuscript. ZH, YF, and MS revised the manuscript. All authors approved the version to be published and agreed to be accountable for all aspects of the work in ensuring accuracy.

### Conflict of interest statement

The authors declare that the research was conducted in the absence of any commercial or financial relationships that could be construed as a potential conflict of interest.
