# Supplementary material for: Transcriptome Comparison Reveals the Adaptive Evolution of Two Contrasting Ecotypes of Zn/Cd Hyperaccumulator Sedum alfredii Hance
Source: Front Plant Sci. 2017 Apr 7;8:425. doi: 10.3389/fpls.2017.00425 (PMC5383727; doi:10.3389/fpls.2017.00425)
Supplement: Supplementary file 1 [file Table1.pdf]

Table S1 Information of primers and resequencing results of random divergent orthologous genes of both HE and NHE.

OG ID	Contig ID	Forward primer	Reverse primer	Product size	No. of inconsistent sites in *Sedum alfredii* Hance
OG04684	HE|c107180_g1	CTAGCGCACCAATTTCTCAGCATGTT	TCAAAGAGTTGCAACTGAACTAGTGTT	567	1
OG04684	NHE|c62757_g1	CACCAATTTCTCAGCATGTTCATGGC	TTAGAGAGATGCAGGTGAAGTAGTGTT	564	0
OG11524	HE|c41849_g1	GACGTTCCAAAAAGATTATCTTTTA	AACATCTTCATCTTCAGCTTGATG	333	0
OG11524	NHE|c75692_g1	TGTCGCAACGTTGCCACGCC	TTTCAACATCTTCATCTTCAGCTTGAT	505	4
OG27948	HE|c28007_g1	GTTTATTACTCCATGTGTTTGGTTCT	ATTAAACTGATCTTGCAACATCCTA	390	0
OG27948	NHE|c69277_g1	GTTTATTACTCCATGTGTTTGGTTC	AGTCATATACCAAAACACCCAAAAGG	309	0
OG29785	HE|c42238_g2	ATCAGCGAACGAACTCAGCGCAAGATT	TTAGAATGCATCTTTAAGGAGTTCC	501	0
OG29785	NHE|c75239_g2	CACGGCTCGGGCTTCGTCTT	CCGGGGGGCTTTCATCATTCTT	762	0
OG30653	HE|c4929_g1	GAGCTCACACATCTAACAGTCCCGA	CTAACCAATTCGAGGAGCTCTGGAA	339	2
OG30653	NHE|c62840_g1	GAGCTCACACATCTAACAGTCCCGA	CTAACCAATTCGAGGAGCTCTGGAA	339	5
OG34157	HE|c87800_g1	ACTTTTTCAGGTGTGATCACAGCTGC	CTCACCCAAATTAGTCATCACATC	279	0
OG34157	NHE|c56126_g1	ACTTTTTCAGGTGTGATCACAGCTGC	CTCACCCAAATTAGTCATCACATC	279	2
OG30137	NHE|c66407_g1	ATGGCGTCCTTTTGCGTTTCGC	GATGGTAACCACAATGTCCTCGTT	753	0
OG30430	NHE|c56724_g1	GGCCATATATCCTCCTTAGAGGAGAG	TTCTTTAGCTTCTTTAGAGATCTTGGC	237	1
OG30580	NHE|c76500_g6	CAAATCCGAGTTCGCCAATCCG	GTCTCCAAGGTTAGCAGCCTCT	180	0
