# Supplementary material for: Transcriptome Comparison Reveals the Adaptive Evolution of Two Contrasting Ecotypes of Zn/Cd Hyperaccumulator Sedum alfredii Hance
Source: Front Plant Sci. 2017 Apr 7;8:425. doi: 10.3389/fpls.2017.00425 (PMC5383727; doi:10.3389/fpls.2017.00425)
Supplement: Supplementary file 2 [file Table2.pdf]

Table S2 Numbers and percentages of unigenes annotation database. HE, hyperaccumulating ecotype of *S. alfredii* Hance; NHE, non-hyperaccumulating ecotype of *S. alfredii* Hance.

Annotation database	No.(%) of HE	No.(%) of NHE
Annotated in NR	62333 (52.61)	126938 (55.66)
Annotated in NT	19087 (16.11)	48460 (21.24)
Annotated in KO	28150 (23.75)	48697 (21.35)
Annotated in SwissProt	49716 (41.96)	91585 (40.15)
Annotated in PFAM	52274 (44.12)	96694 (42.4)
Annotated in GO	54994 (46.41)	104365 (45.76)
Annotated in KOG	35279 (29.77)	67526 (29.61)
Annotated in all Databases	8330 (7.03)	13874 (6.08)
Annotated in at least one Database	73617 (62.13)	149378 (65.5)
Total Unigenes	118479 (100)	228051 (100)
