# Supplementary material for: Transcriptome Comparison Reveals the Adaptive Evolution of Two Contrasting Ecotypes of Zn/Cd Hyperaccumulator Sedum alfredii Hance
Source: Front Plant Sci. 2017 Apr 7;8:425. doi: 10.3389/fpls.2017.00425 (PMC5383727; doi:10.3389/fpls.2017.00425)
Supplement: Supplementary file 3 [file Table3.pdf]

Table S3 Summary of SNP loci information. HE, hyperaccumulating ecotype of *S. alfredii* Hance; NHE, non-hyperaccumulating ecotype of *S. alfredii* Hance.

SNPs forms	No (%). of HE	No (%). of NHE
AG	45264 (30.2%)	98676 (30.9%)
CT	43919 (29.3%)	97192 (30.4%)
Transition	89183 (59.5%)	195868 (61.3%)
AC	14900 (10.0%)	30606 (9.6%)
AT	18517 (12.4%)	39490 (12.3%)
CG	11977 (8.0%)	23193 (7.3%)
GT	15091 (10.1%)	30673 (9.6%)
Transversion	60485 (40.5%)	123962 (38.7%)
All	149668 (100%)	319830 (100%)
