# Supplementary material for: Transcriptome Comparison Reveals the Adaptive Evolution of Two Contrasting Ecotypes of Zn/Cd Hyperaccumulator Sedum alfredii Hance
Source: Front Plant Sci. 2017 Apr 7;8:425. doi: 10.3389/fpls.2017.00425 (PMC5383727; doi:10.3389/fpls.2017.00425)
Supplement: Supplementary file 4 [file Table4.pdf]

Table S4 Summary of SNPs information. HE, hyperaccumulating ecotype of *S. alfredii* Hance; NHE, non-hyperaccumulating ecotype of *S. alfredii* Hance.

Summary of SNPs	No.(%) of HE	No.(%) of NHE
Total SNPs	149668 (100%)	319830 (100%)
Non coding region SNPs	94548 (63.17%)	170673 (53.36%)
Coding region SNPs	55120 (36.83%)	149157 (46.64%)
Synonymous SNPs	54955 (36.72%)	148864 (46.54%)
Nonsynonymous SNPs	165 (0.11%)	293 (0.09%)
