# Supplementary material for: Transcriptome Comparison Reveals the Adaptive Evolution of Two Contrasting Ecotypes of Zn/Cd Hyperaccumulator Sedum alfredii Hance
Source: Front Plant Sci. 2017 Apr 7;8:425. doi: 10.3389/fpls.2017.00425 (PMC5383727; doi:10.3389/fpls.2017.00425)
Supplement: Supplementary file 5 [file Table5.pdf]

Table S5 Summary of SSRs loci information. HE, hyperaccumulating ecotype of *S. alfredii* Hance; NHE, non-hyperaccumulating ecotype of *S. alfredii* Hance.

Summary of SSR	No. of HE	No. of NHE
Monomers	5034	4907
Dimers	2407	2833
Trimers	4878	6258
Quadmers	302	341
Pentamers	26	32
Hexamers	44	57
Total	12691	14428
Unigenes containing SSRs	10746	12393
Unigenes containing >1 SSRs	1579	1653
Compound SSR	629	760
