# Supplementary material for: Transcriptome Comparison Reveals the Adaptive Evolution of Two Contrasting Ecotypes of Zn/Cd Hyperaccumulator Sedum alfredii Hance
Source: Front Plant Sci. 2017 Apr 7;8:425. doi: 10.3389/fpls.2017.00425 (PMC5383727; doi:10.3389/fpls.2017.00425)
Supplement: Supplementary file 6 [file Table6.PDF]

Table S6 Information and annotation of purified orthologous genes

OG	HE unigene	NHE unigene	Ka	Ks	Ka/Ks	p value	annotation
OG32440	HE|c680_g1	NHE|c201178_g1	0.0711	0.9336	0.0762	0.036347	40S ribosomal protein S16, RP-S16e
OG28687	HE|c34502_g2	NHE|c56889_g2	0.0475	1.0854	0.0437	4.53E-05	40S ribosomal protein S23, RP-S23e
OG28534	HE|c33325_g1	NHE|c407_g1	0.0276	1.9799	0.014	0.004731	40S ribosomal protein S7, RP-S7e
OG29877	HE|c42929_g2	NHE|c56212_g1	0.0281	0.4834	0.0582	0.007189	40S ribosomal protein S9, RP-S9e
OG11475	HE|c42260_g1	NHE|c67438_g1	0.0024	0.0889	0.0268	0.014131	50S ribosomal protein L23, RP-L23
OG29172	HE|c38185_g1	NHE|c60520_g1	0.0295	0.3001	0.0984	0.005822	60S ribosomal protein L13, RP-L13e
OG29455	HE|c40105_g1	NHE|c135335_g1	0.0248	2.205	0.0113	2.32E-06	60S ribosomal protein L19, RP-L19e
OG26385	HE|c11756_g1	NHE|c62415_g1	0.0064	1.3499	0.0047	0.011036	60S ribosomal protein L23, RP-L23e
OG28313	HE|c31626_g1	NHE|c116622_g1	0.0188	1.0014	0.0187	0.00466	60S ribosomal protein L26, RP-L26e
OG32171	HE|c6553_g1	NHE|c200509_g1	0.0224	1.5012	0.0149	0.020431	60S ribosomal protein L27, RP-L27e
OG27122	HE|c18511_g1	NHE|c168022_g1	0.0691	0.7242	0.0955	0.000281	60S ribosomal protein L35, RP-L35e
OG31825	HE|c61021_g1	NHE|c37679_g1	0.0146	1.4878	0.0098	0.000771	60S ribosomal protein L37, RP-L37e
OG27694	HE|c25292_g1	NHE|c85053_g1	0.0413	0.6984	0.0591	0.003432	60S ribosomal protein L44, RP-L44e
OG29450	HE|c40085_g1	NHE|c66902_g4	0.0328	0.4886	0.0672	2.68E-06	ADP-ribosylation factor
OG26326	HE|c11668_g1	NHE|c178847_g1	0.0112	1.025	0.0109	0.000118	ADP-ribosylation factor
OG28399	HE|c32317_g1	NHE|c45277_g1	0.0065	0.16	0.0404	0.025428	Autophagy-related protein
OG07011	HE|c45014_g1	NHE|c78255_g1	0.0093	0.1262	0.0735	0.002291	Bifunctional uridylyltransferase
OG32168	HE|c65495_g1	NHE|c102706_g1	0.1747	1.9316	0.0905	0.045872	Cathepsin
OG06003	HE|c53916_g1	NHE|c82819_g1	0.0171	0.1831	0.0932	0.044416	DnaJ homolog subfamily C member
OG10409	HE|c44189_g1	NHE|c75934_g1	0.2494	4.0596	0.0614	0.041724	Dual specificity protein phosphatase

OG26809	HE|c14372_g1	NHE|c174373_g1	0.345	5.8151	0.0593	0.000366	Dynein heavy chain, axonemal
OG28856	HE|c35895_g1	NHE|c80025_g2	0.016	0.2887	0.0553	0.001423	Ethylene-responsive transcription factor
OG32500	HE|c68805_g1	NHE|c66947_g1	0.016	0.179	0.0892	0.04895	Ethylene-responsive transcription factor
OG28008	HE|c28584_g1	NHE|c41007_g1	0.0363	0.5248	0.0692	0.044999	Eukaryotic translation initiation factor
OG25962	HE|c11310_g1	NHE|c64297_g1	0.0349	0.4811	0.0726	0.033851	F-type H+-transporting ATPase
OG26734	HE|c13142_g1	NHE|c106108_g1	0.0537	0.7204	0.0746	0.002084	V-type H+-transporting ATPase
OG34497	HE|c91096_g1	NHE|c4239_g1	0.0735	0.8267	0.0889	0.007102	V-type H+-transporting ATPase
OG34275	HE|c89189_g1	NHE|c180930_g1	0.0695	1.3385	0.0519	0.021685	Glutamine synthetase
OG26418	HE|c118067_g1	NHE|c144258_g1	0.0237	0.9278	0.0255	0.003558	GTP-binding protein SAR1
OG29246	HE|c38673_g1	NHE|c70026_g2	0.0154	0.8982	0.0171	0.000265	Heat shock 70kDa protein
OG29577	HE|c40915_g1	NHE|c174192_g1	0.039	2.6017	0.015	0.016593	High mobility group protein
OG11638	HE|c48618_g1	NHE|c78530_g1	0.012	0.1362	0.0882	0.044091	L-aspartate oxidase
OG33860	HE|c83696_g1	NHE|c74395_g1	0.0533	0.7899	0.0674	0.000583	Lysophospholipid acyltransferase
OG26544	HE|c119957_g1	NHE|c32475_g1	0.0614	1.4117	0.0435	0.002543	Malate dehydrogenase
OG31919	HE|c62150_g1	NHE|c11706_g1	0.0689	0.6987	0.0985	0.045018	Mitogen-activated protein kinase kinase kinase
OG10083	HE|c43201_g1	NHE|c79284_g1	0.0095	0.1834	0.0519	0.040596	NAD(P)H-quinone oxidoreductase
OG30139	HE|c4477_g1	NHE|c52036_g1	0.005	0.0796	0.0628	0.000445	Nascent polypeptide-associated complex subunit beta
OG29791	HE|c42276_g1	NHE|c1773_g1	0.0158	0.1883	0.0839	0.001142	Nitrate transporter
OG05223	HE|c49059_g1	NHE|c81950_g3	0.0154	0.2346	0.0655	0.041279	Novel plant SNARE
OG28815	HE|c35620_g1	NHE|c38067_g1	0.092	1.427	0.0644	0.000108	Nucleoside-diphosphate kinase, E2.7.4.6, ndk
OG33056	HE|c7510_g1	NHE|c104033_g1	0.1276	2.5693	0.0497	0.042993	Peroxiredoxin Q
OG16037	HE|c77465_g1	NHE|c65628_g1	0.0117	0.1526	0.0768	0.02217	Phenylpyruvate tautomerase
OG09796	HE|c51351_g1	NHE|c161244_g1	0.0095	0.1015	0.0935	0.002408	Plastidal glycolate/glycerate translocator

OG06049	HE|c50846_g1	NHE|c78068_g1	0.004	0.071	0.0565	0.04294	Polygalacturonase
OG05035	HE|c46984_g1	NHE|c70322_g1	0.0048	0.0994	0.0482	0.019299	Proline synthase co-transcribed bacterial homolog protein
OG25176	HE|c104190_g1	NHE|c118504_g1	0.0236	0.7527	0.0314	0.045494	Protein transport protein SEC13
OG27933	HE|c27800_g1	NHE|c76410_g5	0.0724	2.1097	0.0343	0.001833	Ras-related C3 botulinum toxin substrate
OG31220	HE|c54736_g1	NHE|c23311_g1	0.0075	0.8516	0.0088	0.005907	Ras-related protein
OG29358	HE|c39402_g1	NHE|c229539_g1	0.009	0.8156	0.011	0.042259	S-phase kinase-associated protein
OG11738	HE|c45934_g1	NHE|c70606_g1	0.0191	0.2079	0.0918	0.045998	Superoxide dismutase [Fe], chloroplastic-like
OG33939	HE|c847_g1	NHE|c64557_g1	0.077	0.7957	0.0967	0.049753	Tartrate-resistant acid phosphatase
OG05036	HE|c7830_g1	NHE|c117042_g1	0.008	0.1536	0.0519	0.033015	Thylakoid lumenal protein
OG34814	HE|c94408_g1	NHE|c66904_g2	0.0335	0.6959	0.0481	0.026781	Triosephosphate isomerase
OG26693	HE|c1247_g1	NHE|c163885_g1	0.0153	1.4985	0.0102	0.007302	Ubiquitin-conjugating enzyme E2
OG28980	HE|c36716_g1	NHE|c59918_g1	0.0105	0.1138	0.0922	0.037206	Vesicle-associated membrane protein
OG34409	HE|c90371_g1	NHE|c12514_g1	0.0095	1.1495	0.0083	0.004113	Y-box-binding protein
OG27110	HE|c182_g1	NHE|c143168_g1	0.0757	2.0854	0.0363	0.022486	Hypothetical protein
