# Supplementary material for: Transcriptome Comparison Reveals the Adaptive Evolution of Two Contrasting Ecotypes of Zn/Cd Hyperaccumulator Sedum alfredii Hance
Source: Front Plant Sci. 2017 Apr 7;8:425. doi: 10.3389/fpls.2017.00425 (PMC5383727; doi:10.3389/fpls.2017.00425)

Figure S1. The logic flow of orthologous genes analysis of *Sedum alfredii* Hance. HE, hyperaccumulating ecotype *S. alfredii* Hance.

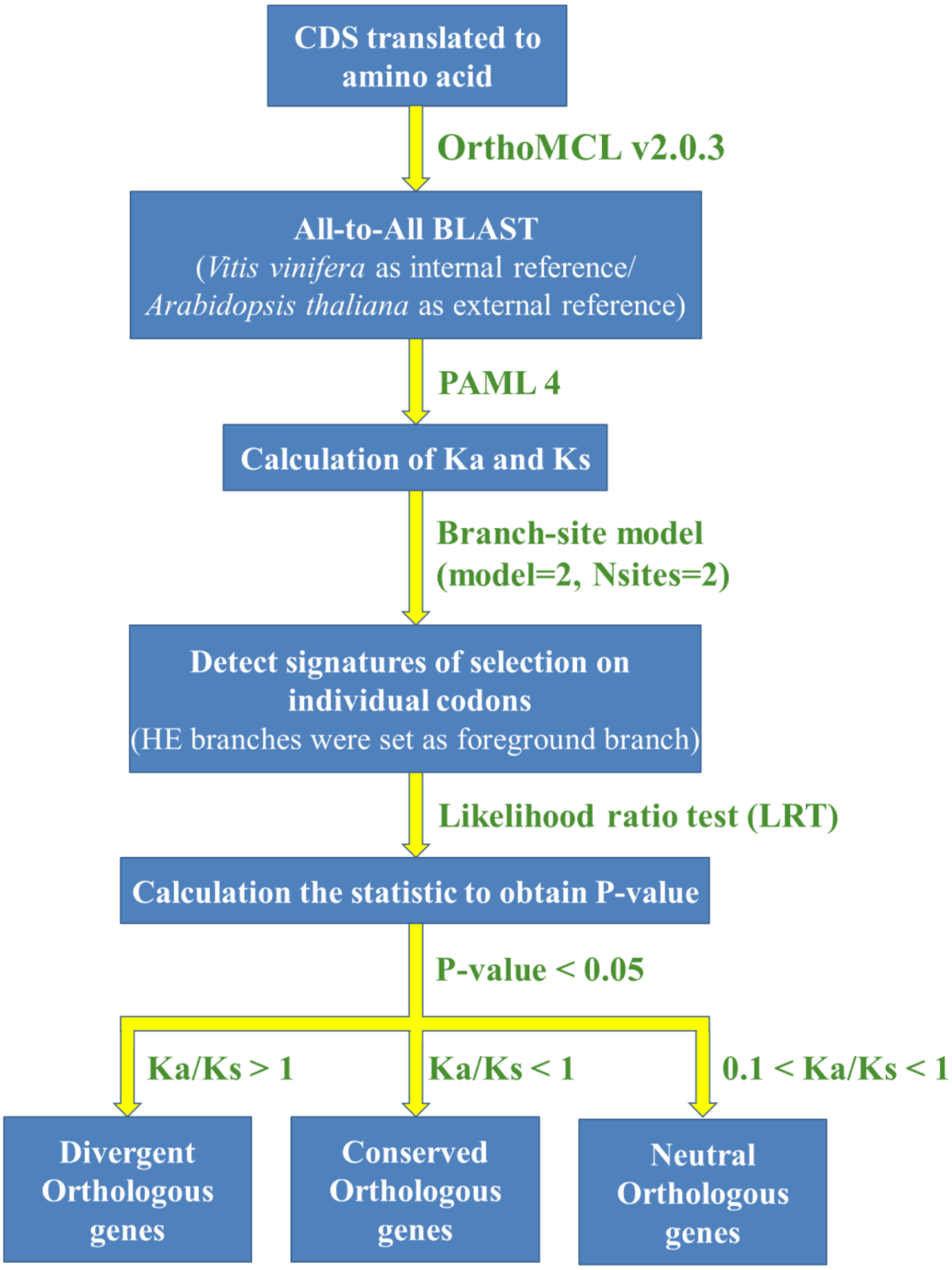

Supplement: Supplementary file 10 [file Image1.PDF]
