# Supplementary material for: Transcriptome Comparison Reveals the Adaptive Evolution of Two Contrasting Ecotypes of Zn/Cd Hyperaccumulator Sedum alfredii Hance
Source: Front Plant Sci. 2017 Apr 7;8:425. doi: 10.3389/fpls.2017.00425 (PMC5383727; doi:10.3389/fpls.2017.00425)

Figure S5. SSR motifs distribution of hyperaccumulating ecotype (HE) (A) and non-hyperaccumulating ecotype (NHE) (B) of *Sedum alfredii* Hance.

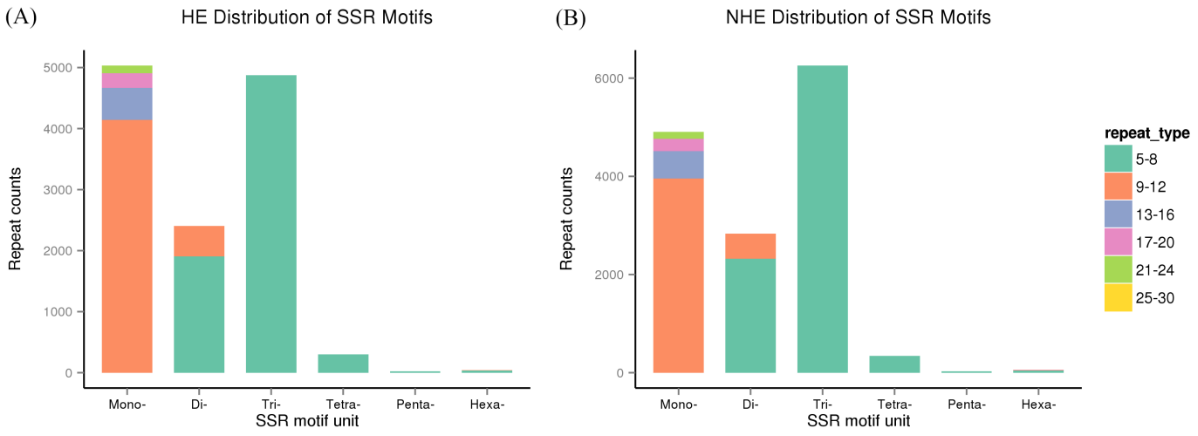

Supplement: Supplementary file 14 [file Image5.PDF]
