# Supplementary material for: Transcriptome Comparison Reveals the Adaptive Evolution of Two Contrasting Ecotypes of Zn/Cd Hyperaccumulator Sedum alfredii Hance
Source: Front Plant Sci. 2017 Apr 7;8:425. doi: 10.3389/fpls.2017.00425 (PMC5383727; doi:10.3389/fpls.2017.00425)

Figure S6. Unigenes Kyoto Encyclopedia of Genes and Genomes (KEGG) of conserved orthologous genes.

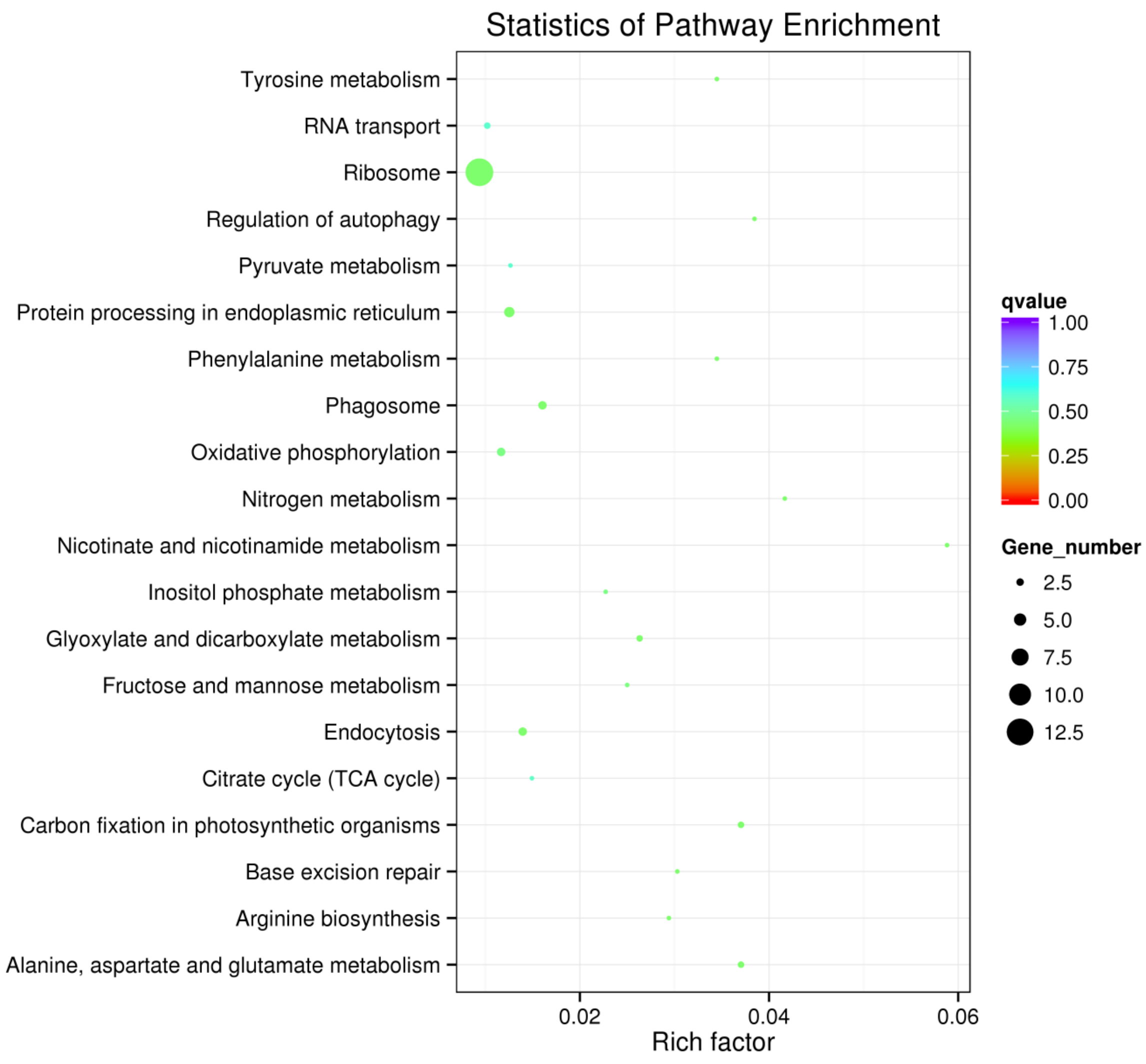

Supplement: Supplementary file 15 [file Image6.PDF]
